# Augmenting the Post-Transplantation Growth and Survivorship of Juvenile Scleractinian Corals via Nutritional Enhancement

**DOI:** 10.1371/journal.pone.0098529

**Published:** 2014-06-04

**Authors:** Tai Chong Toh, Chin Soon Lionel Ng, Jia Wei Kassler Peh, Kok Ben Toh, Loke Ming Chou

**Affiliations:** Reef Ecology Laboratory, Department of Biological Sciences, National University of Singapore, Singapore, Singapore; Smithsonian’s National Zoological Park, United States of America

## Abstract

Size-dependant mortality influences the recolonization success of juvenile corals transplanted for reef restoration and assisting juvenile corals attain a refuge size would thus improve post-transplantation survivorship. To explore colony size augmentation strategies, recruits of the scleractinian coral *Pocillopora damicornis* were fed with live *Artemia salina* nauplii twice a week for 24 weeks in an *ex situ* coral nursery. Fed recruits grew significantly faster than unfed ones, with corals in the 3600, 1800, 600 and 0 (control) nauplii/L groups exhibiting volumetric growth rates of 10.65±1.46, 4.69±0.9, 3.64±0.55 and 1.18±0.37 mm^3^/week, respectively. Corals supplied with the highest density of nauplii increased their ecological volume by more than 74 times their initial size, achieving a mean final volume of 248.38±33.44 mm^3^. The benefits of feeding were apparent even after transplantation to the reef. The corals in the 3600, 1800, 600 and 0 nauplii/L groups grew to final sizes of 4875±260 mm^3^, 2036±627 mm^3^, 1066±70 mm^3^ and 512±116 mm^3^, respectively. The fed corals had significantly higher survival rates than the unfed ones after transplantation (63%, 59%, 56% and 38% for the 3600, 1800, 600 and 0 nauplii/L treatments respectively). Additionally, cost-effectiveness analysis revealed that the costs per unit volumetric growth were drastically reduced with increasing feed densities. Corals fed with the highest density of nauplii were the most cost-effective (US$0.02/mm^3^), and were more than 12 times cheaper than the controls. This study demonstrated that nutrition enhancement can augment coral growth and post-transplantation survival, and is a biologically and economically viable option that can be used to supplement existing coral mariculture procedures and enhance reef restoration outcomes.

## Introduction

The global decline of coral reefs and the loss of associated ecological services have necessitated immediate intervention measures to reverse their further deterioration [1], . Active coral reef restoration initiatives have increasingly been incorporated into coastal management frameworks to supplement existing measures of rehabilitating impacted reefs [3], [4]. Of the myriad techniques which have been developed, coral transplantation remains one of the most widely used, largely due to its ability to promote rapid colonization of the reefs and its ease of application [3], [5]. The potential for generating large quantities of coral material via the “coral gardening” approach [6] for eventual transplantation to degraded reefs led to a greater emphasis on coral mariculture techniques. Asexual propagation techniques such as fragmentation allow coral material to be generated easily [3], but the drawbacks of this approach include a lack of genetic diversity of the clonal fragments and susceptibility of the donor colonies to stress arising from the fragmentation process, hence impeding large-scale production [7], [8], [9]. Recent developments have enabled the use of sexually derived coral juveniles as material for transplantation onto degraded reefs [10], [11]. As scleractinian corals are highly fecund, this ensures that large numbers of genetically diverse coral propagules would be generated. While direct artificial seeding of coral larvae onto reefs can enhance initial recruitment [12], early post-settlement mortality of the recruits is exceedingly high due to competition by fouling communites and predation [13].

The use of *ex situ* coral mariculture in reef restoration can improve coral post-settlement survivorship. The rearing conditions can be carefully monitored and regulated to minimize the impacts of disturbances from fouling communities, temperature fluctuations and predator infestations by allowing the timely introduction of mitigative measures [14], [15]. In spite of these benefits, the cost of setting up and operating *ex situ* mariculture facilities can be very expensive [4]. For instance, the cost of maintaining juvenile coral culture in the Philippines for six months constitutes 42.9% of the total project expenditure [11] and this inevitably increases with labour costs [16]. Unfortunately, such detailed financial estimates are rarely reported in the existing scientific literature due to the complexities involved and the rigorous efforts required to provide a reliable estimate. Cost-effectiveness analyses of cost-per-coral reveal clearly that as mortality rate increases, so does the cost of each colony [4]. Given that the highest mortality rates occur during the early developmental phases of the coral life cycle [13], augmenting the survivorship of juvenile corals would improve cost-effectiveness and increase the availability of source material for transplantation.

Size is an important determinant of survivorship in scleractinian corals and thus affects the rate of establishment of coral transplants on degraded reefs [17]. Smaller colonies tend to be more vulnerable since the refuge size required for surviving injuries arising from predation and incidental grazing is not yet attained [15], [18]. Increasing coral colony size prior to transplantation is thus advantageous for enhancing post-transplantation growth, survivorship and promoting sexual maturity – factors which are essential for the maintenance of a viable coral community [17], [19], [20].

Scleractinian corals exhibit substantial inter- and intra-specific variations in growth rates [19], [21], and one potential approach to promote rapid colony growth is to facilitate colony fusion [17]. However tissue resorption and somatic germ-cell parasitism may instead retard colony growth [22], [23], [24]. Another strategy involves enhancing the autotrophic and heterotrophic modes of coral nutrition by adjusting the conditions in *ex situ* mariculture prior to transplantation. Various studies have demonstrated that photosynthetic and feeding rates could be increased by the manipulation of light intensity, flow rate and nutrient levels [25], [26], [27]. Although information on the effects of these manipulations on long-term coral growth rates is limited, the effects of nutritional enhancement are remarkably consistent for coral species from the families Faviidae, Acroporidae and Pocilloporidae. Compared to non-live feeds, live feeds were particularly useful for inducing faster coral growth [28], as were increments in *ex situ* feeding densities [28], [29]. With heterotrophy in scleractinian corals commencing as early as two to seven days post-settlement [30], [31], enhancing nutrition in the early stages should be explored as this would assist coral juveniles in attaining a size refuge as early as possible and reduce mortality.

The present study aims to evaluate the feasibility of nutritional enhancement as a strategy to improve the post-transplantation growth and survivorship of juveniles of the scleractinian coral *Pocillopora damicornis*. We hypothesize that the growth and survivorship of fed corals would be augmented both during the *ex situ* mariculture phase and after transplantation to the reef. To assess the economic viability of this approach for both coral mariculture and reef restoration efforts, we also determined the cost estimates for the study and examined the cost-effectiveness of *ex situ* nutritional enhancement. The findings of this study will facilitate planning of future coral mariculture and reef restoration initiatives.

## Materials and Methods

### Study Species and Planulae Collection

*Pocillopora damicornis* (Linnaeus, 1758) is a hermaphroditic scleractinian coral commonly found inhabiting shallow coastal areas within the Indo-Pacific region [32]. The reproductive and feeding biology of this coral has been well-studied [31], [33], [34] and it has been used extensively as a model species for developmental studies [17]. *Pocillopora damicornis* is also highly fecund and broods monthly [35], making it a popular candidate for propagation for the aquarium trade and reef restoration. This research was conducted with permission from Singapore National Parks Board (permit number NP/RP13-016), and no permit is required for collecting coral propagules in Singapore. Ten donor colonies of *P. damicornis* were collected from the fringing reef off Kusu Island, Singapore (1°13′25′′N, 103°51′38′′E) two days before the new moon in July 2012. Only colonies spaced 5 m apart and at least 20 cm in diameter were collected to ensure that they were sexually mature and to minimize the chances of collecting identical genets [36]. The colonies were then transported to the Tropical Marine Science Institute on St. John’s Island, Singapore (1°13′44′′N, 103°50′73′′E) and maintained in aerated outdoor aquaria (190×100×40 cm) with flow-through filtered seawater [9], which functioned as an *ex situ* coral nursery.

Biologically conditioned ‘plugs’, made of plastic wall plugs embedded in cement hemispheres (40 mm diameter), were fabricated and used as settlement substrates [15]. These allowed the *P. damicornis* recruits to be handled easily and facilitated their eventual transplantation onto the reef. One day before the new moon, all donor colonies were transferred and isolated in polyethylene planulation tanks (45 cm×30 cm×30 cm) with flow-through filtered seawater. Five centimetres below the rim of each tank, an outlet (3 cm diameter) was created to ensure water exchange. It was also covered with 100 µm plankton mesh (Sefar Pte. Ltd., Singapore) which helped to retain the coral planulae within the tanks. The tanks were filled with approximately 8 cm of sand which the conditioned plugs were inserted into, leaving only their hemispherical surfaces exposed for planulae settlement. Each plug was monitored daily for newly settled recruits, and plugs with at least three recruits were removed from the tank and maintained in the outdoor aquaria. In this study, all the colonies planulated within one to six days after the new moon, and the planulae were observed to settle within a day after planulation.

### Feeding Regime in *ex situ* Coral Nursery

A total of 288 plugs with live juvenile corals were used for this study. On each plug, one primary polyp which had settled at least 10 mm away from the rest of the recruits was identified, measured and tagged by mapping the coral’s position on the plug. This served to reduce the chances of colony fusion which would affect growth rates [17]. The plugs were randomly assigned among 16 holding tanks, each tank corresponding to one of the four feeding densities (0, 600, 1800 and 3600 nauplii/L following Petersen et al. (2008); *n* = 4 tanks). In each replicate tank, 18 plugs spaced 5 cm apart were secured on an elevated PVC frame. All plugs were maintained in the outdoor aquaria for one week before the start of the feeding regime [28].

The juvenile corals were fed with cultured day-old *Artemia salina* (approximately 400 µm; Bio-Marine Inc., California, U.S.A.), wherein each nauplius provided around 9.77 µcal [37], for 4 hours (between 12∶00 to 16∶00) twice every week for 24 weeks (from August 2012 to February 2013). During each feeding session, all the plugs were transferred to 10 L polyethylene feeding tanks containing 6 L of filtered sea water with gentle aeration. The corresponding volume of nauplii stock solution was added to make up the required densities for each treatment tank. The positions of the feeding tanks were randomised during each feeding session to minimize potential spatial influences on heterotrophic rates. After feeding, the plugs were gently flushed with filtered seawater to remove any remaining nauplii, and subsequently transferred back to the holding tanks. Fouling macroalgae were physically removed twice a week as these would otherwise rapidly overgrow the coral juveniles and compromise colony health [15].

The survivorship and growth – length (*l*), width (*w*) and height (*h*) – of the 18 tagged coral juveniles in each replicate tank were measured using vernier calipers every four weeks and the ecological volume of each coral was estimated following the calculation for right cylindrical volumes, *V* = *πr^2^h*, where *r* = (*l+w*)/4 [38]. Weekly radial and volumetric growth were calculated by dividing the respective differences in colony radii and ecological volumes at the start of the *ex situ* feeding regime and at the end of the *ex situ* feeding regime (24 weeks). The data obtained for all the surviving corals in each replicate tank was then averaged. The mean daily temperature and light irradiance (Onset Computer Corporation Inc., Massachusetts, U.S.A.) in the aquaria were 29±0.01°C (*n* = 168 days) and 128.7±18.1 Lux (*n* = 168 days) respectively.

### Transplantation and Monitoring

After 24 weeks, eight plugs with live corals were randomly selected from each holding tank to be transplanted back to the donor reef at Kusu Island. Four limestone outcrops (approximately 3.5 m in diameter and 2.5 m in height) that were at least 5 m apart were identified for the transplantation of the juvenile corals. Four sets of eight holes were then drilled on each outcrop and each replicate treatment was randomly assigned to one set of holes, such that the corals belonging to the same replicate holding tank were transplanted on the same outcrop (*n = *4 outcrops). The plugs were inserted into the holes and stabilized using two-part marine epoxy [11].

The survivorship and colony dimensions of the tagged coral juveniles on each plug were recorded every four weeks for 24 weeks (from February to August 2013). Weekly radial and volumetric growth were calculated by dividing the respective differences in colony radii and ecological volumes at the start of the *ex situ* feeding regime and at the end of the entire study with the duration of the entire study (48 weeks). The data obtained for all the surviving corals in each replicate outcrop was then averaged. The mean daily temperature (Onset Computer Corporation Inc., Massachusetts, U.S.A.) in the transplant site was 29.9±0.07°C (24 readings per day, *n = *168 days).

### Cost Analysis

Cost-estimates were tabulated for each of the five phases of this study: (1) Collection of source materials and establishment of coral culture, (2) Maintenance and *ex situ* monitoring, (3) Feeding, (4) Transplantation and (5) *In situ* monitoring, and further itemized into equipment costs, labour costs and boat trips following Edwards et al. (2010) and Villanueva et al. (2012). The cost per coral produced before and after transplantation to the reef were then calculated. In addition, the cost per unit volumetric growth of each treatment group for both the *ex situ* feeding and post-transplantation phases was also estimated based on the total production costs, the mean weekly volumetric growth rates, duration and the number of tagged colonies alive at the end of each phase.

### Statistical Analysis

Data for the final ecological volume, weekly radial and volumetric growth rates were first tested for homogeneity of variances using Levene’s test and normality using Shapiro-Wilk test, followed by one-factor ANOVA with Tukey’s HSD (Honestly Significant Difference) post-hoc test for all possible pairwise comparisons. As the variances for post-transplantation volumetric growth rates were heterogenous and not normally distributed, a non-parametric Kruskall-Wallis analysis was used. Subsequent pairwise comparisons were analysed using Mann-Whitney U test. These analyses were computed using SPSS v 17.0 (SPSS Inc). Data for the survivorship was analyzed using Cox Proportional-Hazards regression model and logrank test (R 2.14.2), using the independent factors initial colony radius, treatment and the interaction between radius and treatment for analysis. The model that best explained the trend was then selected using Akaike Information Criteria (AIC).

## Results

### Growth of *Pocillopora damicornis* Juveniles in *ex situ* Feeding Phase

**Figure 1:**
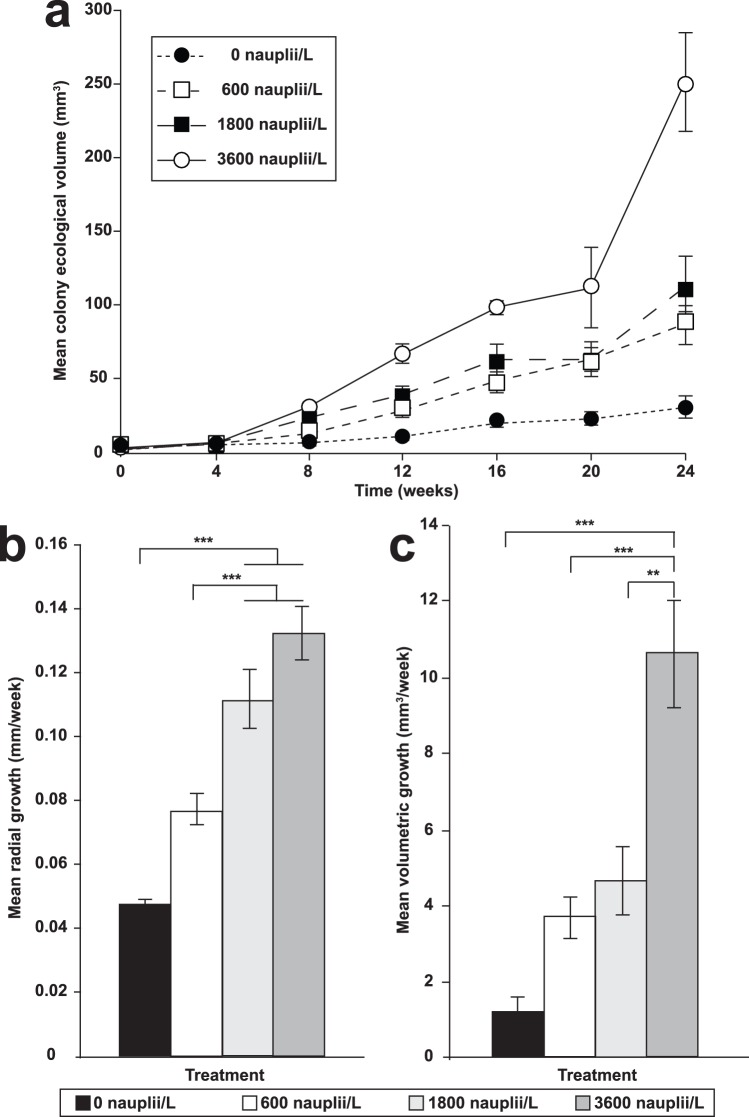
Growth of *Pocillopora damicornis* juveniles over a 24-week *ex situ* feeding regime. Graphs show the (a) mean ecological volumes, (b) mean weekly radial and (c) volumetric growth rates (± S.E.) of the corals in the 0 (control), 600, 1800 and 3600 nauplii/L treatment groups. The symbols *, **, and *** denote statistical significance at *p = *0.05, *p = *0.01, *p = *0.001 respectively.

**Figure 2:**
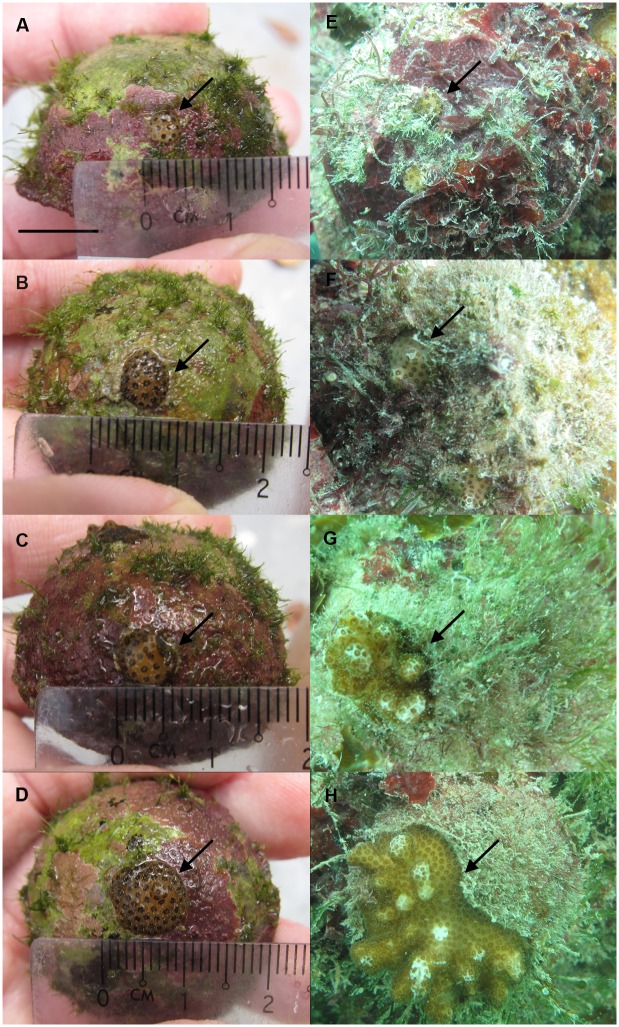
Growth of *Pocillopora damicornis* juveniles throughout the study. *Pocillopora damicornis* juveniles in the 0 (control), 600, 1800 and 3600 nauplii/L treatment groups (a, c, e, g) after the 24-week *ex situ* feeding regime, and (b, d, f, h) 24 weeks after transplantation to the reef. Scale bar = 1 cm, arrows indicate the positions of the corals.

The initial mean colony volume of the coral juveniles (approximately 3.5 mm^3^) did not differ significantly among the treatment groups (*F_3,12_* = 0.687, *p* = 0.557). The mean colony volume across the treatments increased monotonically over the *ex situ* feeding phase of the study (Fig. 1a; Fig. 2). Juvenile corals in the 3600 nauplii/L treatment group grew by more than 74 times their initial sizes and attained a mean final ecological volume of 248.38±33.44 mm^3^ (mean ± S.E.; 4.03±0.18 mm radius). The final volumes of the colonies in the 1800, 600 and 0 nauplii/L were 111.66±20.8 mm^3^ (34 times the initial volume; 3.63±0.25 mm radius), 87.18±12.91 mm^3^ (24 times the initial volume; 2.78±0.12 mm radius) and 30.65±8.65 mm^3^ (8 times the initial volume; 2.13±0.05 mm radius), respectively.

The weekly radial growth rates of the colonies (Fig. 1b) significantly differed among treatments (*F_3,12_* = 30.8, *p*<0.001). Colonies in the 3600 and 1800 nauplii/L treatment groups grew at rates of 0.13±0.008 mm/week (mean ± S.E.) and 0.11±0.009 mm/week respectively, and were significantly faster than those in the 600 nauplii/L (0.08±0.005 mm/week, *p*<0.001) and control (0.05±0.002 mm/week, *p*<0.001) groups. Weekly volumetric growth rates (Fig. 1c) were also significantly different among treatments (*F_3,12_* = 19.2, *p*<0.001) with the colonies in the 3600 nauplii/L treatments growing significantly faster (10.65±1.46 mm^3^/week) than colonies in the 1800 nauplii/L (4.69±0.9 mm^3^/week, *p* = 0.003), 600 nauplii/L (3.64±0.55 mm^3^/week, *p* = 0.001) and control (1.18±0.37 mm^3^/week, *p*<0.001) groups.

### Growth of *Pocillopora damicornis* Juveniles after Transplantation

**Figure 3:**
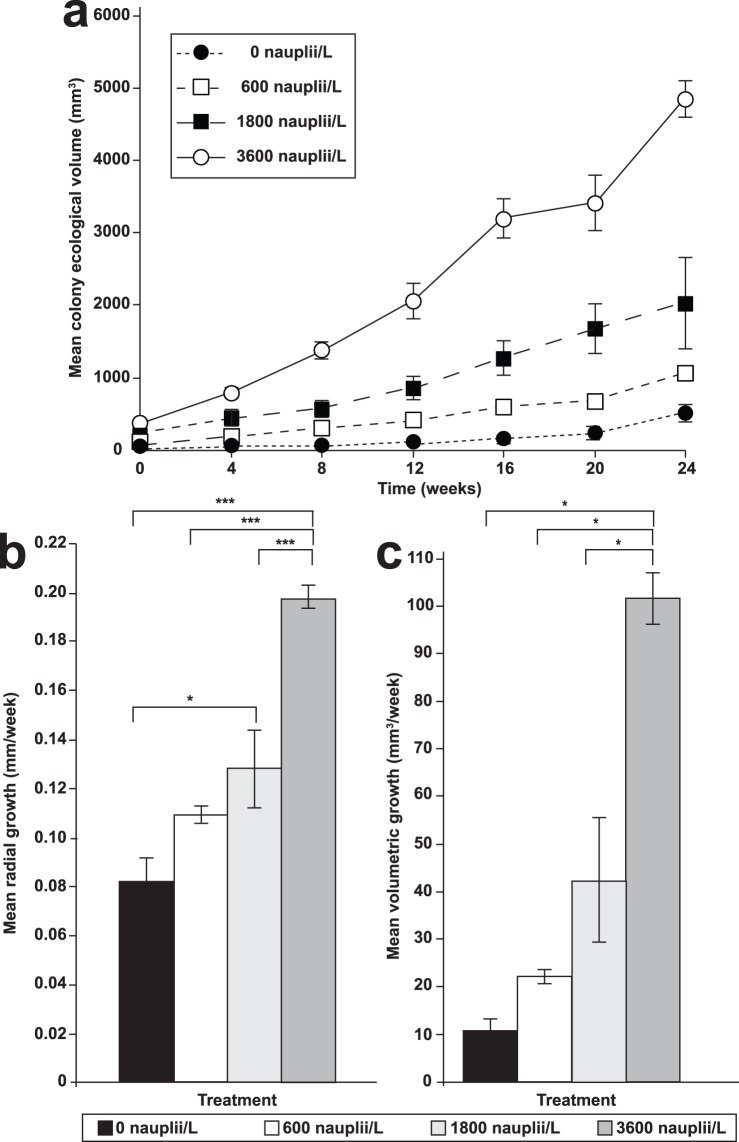
Growth of *Pocillopora damicornis* juveniles over 24 weeks after transplantation to the reef. Graphs show the (a) mean ecological volumes, (b) mean weekly radial and (c) volumetric growth rates (± S.E.) of juvenile *Pocillopora damicornis* in the 0 (control), 600, 1800 and 3600 *Artemia* nauplii/L treatment groups. The symbols *, **, and *** denote statistical significance at *p = *0.05, *p = *0.01, *p = *0.001 respectively.

The mean colony sizes of all juvenile corals continued to increase steadily after transplantation to the reef (Fig. 2; Fig. 3a), with the colonies in the 3600 nauplii/L treatment group exhibiting the largest increase in size (1534 times the initial size at the start of the study). Final mean colony volumes for the 0, 600 1800 and 3600 nauplii/L groups were 512±116 mm^3^ (mean ± S.E.; 137 times the initial volume; 5.03±0.49 mm radius), 1066±70 mm^3^ (284 times the initial volume; 6.35±0.14 mm radius), 2036±627 mm^3^ (486 times the initial volume; 7.25±0.80 mm radius) and 4875±260 mm^3^ (10.5±0.29 mm radius), respectively.

Weekly radial growth rates (Fig. 3b) differed among the treatment groups (*F_3,12_* = 26.05, *p*<0.001). Colonies in the 3600 nauplii/L group (mean ± S.E.; 0.198±0.005 mm/week) had significantly faster growth rates than the colonies in the 1800 nauplii/L (0.128±0.016 mm/week; *p* = 0.001), 600 nauplii/L (0.109±0.003; *p*<0.001) and the control (0.082±0.01 mm/week; *p*<0.001) groups. A significant difference between the 1800 nauplii/L and control groups was also present (*p<*0.05). The weekly volumetric growth rates (Fig. 3b) were also significantly different (*p* = 0.006), displaying a similar trend as that of the radial growth rates. The mean volumetric growth rates (Fig. 3c) were 101.5±5.4 mm^3^/week, 42.3±13.1 mm^3^/week, 22.1±1.5 mm^3^/week and 10.6±2.4 mm^3^/week for the 3600, 1800, 600 and 0 nauplii/L groups respectively.

### Survivorship of Juvenile *Pocillopora damicornis* in *ex situ* Feeding Phase and after Transplantation

**Figure 4:**
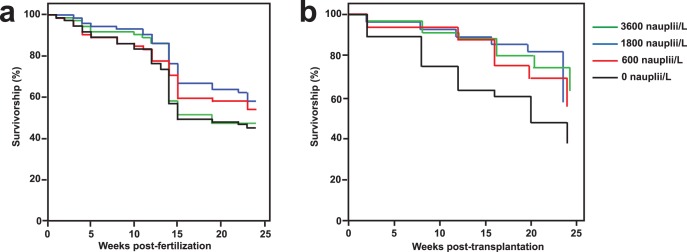
Survivorship of *Pocillopora damicornis* juveniles. Survival curves of *Pocillopora damicornis* juveniles in the 0 (control), 600, 1800 and 3600 nauplii/L groups (a) in the *ex situ* feeding phase (24 weeks, *n* = 72) and (b) after transplantation (24 weeks, *n* = 32).

In the *ex situ* feeding phase (Fig. 4a), there were no significant differences in survivorship across treatments (logrank test = 1.22, *d.f. = *1, *p = *0.27). Survival rates of the *P. damicornis* juveniles in the control, 600, 1800 and 3600 nauplii/L groups at the end of 24 weeks were 45%, 54%, 58% and 47% respectively, and the overall survival was 51%. Corals in the control, 600, 1800 and 3600 nauplii/L groups had post-transplantation survival rates of 38%, 56%, 59% and 63% respectively (overall survival of 54%), and these were significantly different across treatments (Fig. 4b).

Post-hoc pairwise comparisons revealed that the corals in the 3600 and 1800 nauplii/L groups (*p = *0.016 and *p* = 0.044, respectively) had significantly higher survival rates than the control. The difference in survivorship was accounted for by both the initial radius prior to transplantation (logrank test = 6.86, A.I.C. value = 535, *d.f. = *1, *p = *0.009) and treatment (logrank test = 6.26, A.I.C. value = 536, *d.f. = *1, *p = *0.012), and both factors were highly correlated (r = 0.6).

### Cost Analysis

The total cost for producing 288 coral plugs in the *ex situ* feeding phase and transplanting 128 corals to the reef was an estimated US$10467 (see Table S1 for detailed cost estimates). Over 40% was attributed to the cost of establishing the coral culture, which included the harvesting of donor colonies, setting up of culture tanks and the collection of planulae (Table 1). 34.3% of the total costs arose from transplanting and subsquent monitoring of the coral transplants, while feeding and maintenance of the coral juveniles contributed the remaining 9.6% and 7.2% respectively.

**Table 1:** Summary of the cost estimates.

Phase	Subcategory	Cost (US$)	Percentage of total cost (%)
**1. Establishment of coral culture**		**4261.69**	**40.7**
**2. Maintenance**		**757.22**	**7.2**
**3. Feeding regime**		**1000.9**	**9.6**
	3.1 Control treatment	0	0
	3.2 600 nauplii/L	333.27	3.2
	3.3 1800 nauplii/L	333.58	3.2
	3.4 3600 nauplii/L	334.05	3.2
**4. Transplantation**		**860.24**	**8.2**
**5. ** ***In situ*** ** monitoring**		**3587.70**	**34.3**
Grand Total		10467.75	
*Ex situ* production cost for 288 coral plugs		6019.81	
*Ex situ* production cost per coral		20.90	
Cost per coral (51% survival)		40.98	
Cost per coral transplanted (128 coral plugs)		81.78	
Cost (54% survival)		151.44	

Summary of the cost estimates of producing 288 plugs with live *Pocillopora damicornis* juveniles under four ex situ feeding regimes (0, 600, 1800, 3600 nauplii/L) for 24 weeks, followed by the transplantation of 128 coral plugs and subsequent monitoring for 24 weeks. Mean survival rates across the treatments were used for the calculation of cost effectiveness at the end of each phase. Costs were estimated in Singapore Dollars (S$) prior to conversion to US$ at the rate of S$ 1.26 = US$ 1.

The cost of propagating 288 corals was estimated at US$20.90/coral. Upon taking into account the mean survival rate of 51% at the end of the *ex situ* feeding phase, the cost per coral was US$40.98 (Table 1). The cost of each transplanted coral was estimated at US$81.78. With a 54% mean survival rate 24 weeks after transplantation, the cost per coral was US$151.44 (Table 1). In the *ex situ* feeding phase, the cost per unit growth decreased with increasing feeding densities (Table 2), making the 3600 nauplii/L treatment group the most cost-effective. The cost per unit volumetric growth was US$0.18/mm^3^, which was more than seven times cheaper than that of the control group. A similar trend was observed for the corals after transplantation – the cost per unit volumetric growth for the 3600 nauplii/L treatment was US$0.02/mm^3^, which was more than 12 times cheaper than the control treatments.

**Table 2:** Estimated cost per unit volumetric growth of the *Pocillopora damicornis* colonies.

Phase	Treatmentdensity (nauplli/L)	Mean volumetric growth rates (mm^3^/week)	Survival (%)	Estimated total volumetric growth (mm^3^)	Production cost (US$)	Cost per unit volumetric growth (US$/mm^3^)
*Ex situ* feeding	0	1.18	45	3670	5018.91	1.37
	600	3.64	54	13586	6351.99	0.47
	1800	4.69	58	18802	6353.11	0.34
	3600	10.65	47	34598	6355.11	0.18
Transplantation	0	10.6	38	24748	9466.85	0.38
	600	22.1	56	76038	10799.93	0.14
	1800	42.3	59	153335	10801.17	0.07
	3600	101.5	63	392878	10803.05	0.03

Cost per unit volumetric growth of the *Pocillopora damicornis* colonies after the *ex situ* feeding (24 weeks, *n = *288) and transplantation phase (24 weeks, *n* = 128). Total volumetric growth for each phase was estimated based on the mean weekly volumetric growth rates, duration and the number of live tagged colonies at the end of each phase. Production cost for each treatment group was calculated based on the cost estimates for the entire study.

## Discussion

Scleractinian corals supplement up to 35% of their daily metabolic requirements with a wide range of items such as dissolved organic matter, suspended particulate matter and zooplankton [29], [39], [40]. While corals reared in ex situ systems are routinely supplied with zooplankton, microalgae and commercial dry food [28], those fed with live zooplankton – a highly nutritious feed – consistently grow faster [28], [39]. The use of *Artemia* nauplii as coral feed in this study significantly augmented the growth of *P. damicornis* juveniles. Coral volumetric growth rates increased by up to 9 times with the addition of higher densities of *Artemia* nauplii, leading to final ecological volumes that were 2.9 to 8.8 times greater than those in the control groups after 24 weeks (Fig. 1). These results were comparable to work by Petersen et al. (2008), who reported that *Acropora tenuis* juveniles fed with 3750 *Artemia* nauplii/L and *Favia fragum* juveniles fed with 300 nauplii/L respectively grew eight and five times larger than those in the control group. Since juvenile coral growth was proportionate to feed densities and the growth rates did not slow down even at 3600 nauplii/L, further increment of feeding densities and frequency would likely augment coral growth further. Additionally, as heterotrophy is known to play an important role in mitigating effects during stress events such as coral bleaching [41], introducing live feed during the early life stages can assist juvenile corals in attaining the required refuge size faster and cope with the effects of acute environmental stress.

Although *ex situ* mariculture can help to enhance the survivorship of coral fragments (>98%) [42], [43] and sexual propagules (60–75%) [15], [16], [28] by providing a conducive environment for the coral material to grow, the facilities are usually expensive to run [6], inadvertently placing limits on the duration of rearing as well as the potential for any improvements to survivorship [28]. As survivorship increases with colony size [44], it is important to explore ways of accelerating the growth of juvenile corals in the least possible time. In this study, coral survivorship did not improve substantially despite significant increases in growth, as was consistent with that observed by Petersen et al. (2008). However, at 51%, the mean survival rate across treatments were more than four times higher than if juvenile corals of the same size class were to be transplanted to the field [17], underscoring the usefulness of feeding corals in *ex situ* mariculture to optimise restoration outcomes.

Twenty-four weeks after transplantation, the juvenile coral transplants were 1.5 to 2.1 times larger than their initial sizes (Fig. 3). This corroborated with other studies wherein 6-months-old and 18-months-old branching juvenile corals grew 1.5 to five times their initial diameters six months after transplantation [10], [17]. More importantly, the growth rates of fed corals remained consistently higher than those of the unfed corals even after transplantation to the reef, suggesting the possibility that benefits obtained from the *ex situ* feeding regime will continue even after feeding has stopped.

Interestingly, the enhancement in growth from the *ex situ* feeding regime improved the post-transplantation survivorship of the juvenile corals. Both size and feeding regimes were able to account for the survivorship patterns observed, supporting the observations of size-dependant mortality in scleractinian corals [17]. Since nutrition enhancement was a direct causative agent of the coral growth and both the effects of size and feeding regime on survivorship were highly correlated, it exerted a concomitant effect of augmenting post-transplantation survival. Clearly, size was a key determinant of post-transplantation survival. However, the average post-transplantation mortality rate of all *P. damicornis* juveniles in this study (46%) was higher than that reported from other studies (11–34%) [10], [11], [17], likely due to the high sediment levels in Singapore waters, which have been estimated to limit scleractinian recruitment to two individuals m^−2^ [45]. As was observed during monthly visits to the study site, most juvenile colonies were smothered by fine particulate sediment, with obvious damage to the coral tissue. Post-transplantation survivorship can thus be expected to be lower in areas experiencing chronic sedimentation such as Singapore. It is clearly advantageous to boost the survival chances of juvenile corals by implementing an *ex situ* nutritional enhancement regime to increase colony size prior to transplantation.

While nutritional enhancement confers significant ecological advantages to juvenile corals in *ex situ* mariculture, the process should still be thoroughly assessed and reviewed to boost its economic viability. In the current study, nutritional enhancement constituted only 9% of total production costs. Of this amount, 99% was attributed to the labour required for transferring the corals from the holding tanks to the feeding tanks. Such costs can be reduced further in commercial mariculture systems where the corals do not need to be transferred elsewhere for feeding. The results also showed that corals supplied with the highest density of feed (3600 nauplii/L) attained ecological volumes close to that of the corals in the control group at the end of the 24-week feeding phase, in as early as eight weeks. This corresponds to a one-third reduction in *ex situ* rearing time and translates to significant reductions in operational costs. Additionally, the cost-effectiveness of the method was apparent as the cost per unit volumetric growth of the corals fed with 3600 nauplii/L was more than seven and twelve times cheaper than the controls in both the *ex situ* rearing and post-transplantation phases, respectively. However, it must be noted that directly comparing project costs among localities leads to inaccuracies. For example, costs per coral can be as low as US$11 in the Philippines [11] to as high as US$151 in Singapore (this study), mainly due to differences in manpower and equipment costs – labour costs differed by almost six-fold while the cost of boat hire differed by nearly ten-fold. Exploring other options such as recruiting volunteers to reduce labour costs [10] or increasing production for economies of scale [16] would help to improve cost-effectiveness.

The current study showed that supplying live *Artemia salina* nauplii as coral feed enhanced juvenile coral growth rates and survivorship in both the *ex situ* nursery phase as well as six months after they had been transplanted to a reef. These findings are important, because even though sexually-derived corals are increasingly used as material for reef restoration [10], [11], the high mortality rates of the juvenile propagules is often a stumbling block in such projects. Since long rearing periods are infeasible due to high operational costs, nutritional enhancement may be considered as a means of reducing the time and cost required for the coral material to be reared in mariculture facilities. The approach is simple, cost-effective, and harbours the potential for large-scale application.

## Supporting Information

Table S1 **Detailed cost estimates.** Cost estimates of producing 288 plugs with live *Pocillopora damicornis* juveniles under four ex situ feeding regimes (0, 600, 1800, 3600 nauplii/L) for 24 weeks, followed by the transplantation of 128 coral plugs and subsequent monitoring for 24 weeks.
(DOC)
