# Supplementary material for: Augmenting the Post-Transplantation Growth and Survivorship of Juvenile Scleractinian Corals via Nutritional Enhancement
Source: PLoS One. 2014 Jun 4;9(6):e98529. doi: 10.1371/journal.pone.0098529 (PMC4045716; doi:10.1371/journal.pone.0098529)
Supplement: Table S1 — Detailed cost estimates. Cost estimates of producing 288 plugs with live Pocillopora damicornis juveniles under four ex situ feeding regimes (0, 600, 1800, 3600 nauplii/L) for 24 weeks, followed by the transplantation of 128 coral plugs and subsequent monitoring for 24 weeks. (DOC) [file pone.0098529.s001.doc]

**Table S1. Detailed cost estimates.**

|  |  |  | **Time input by personnel** | | | **Dive Gear Hire** | | **SCUBA Air Tank** | | **Boat Time** | | **Sub-total (US$)** | **% of total cost** |
| --- | --- | --- | --- | --- | --- | --- | --- | --- | --- | --- | --- | --- | --- |
| **Phase** | **Activity** | **Equipment cost (US$)** | **Person-hours (h)** | **Rate (US$/h)** | **Costs (US$)** | **Units** | **Costs**  **(US$)** | **Units** | **Costs**  **(US$)** | **Days** | **Costs**  **(US$/day)** |  |  |
| **1. Establishment of coral culture** |  |  |  |  |  |  |  |  |  |  |  | **4261.69** | **40.7** |
|  | 1.1 Collection of source material | 33.33 | 4 X 2 people | 11.90/6.94* | 75.36 | 2 | 55.51 | 2 | 19.05 | 1 | 436.51 | 619.81 | 5.9 |
|  | 1.2. Setting up of holding tanks | 2556.48 | 2 X 2 people | 11.90/6.94* | 37.68 | 0 | 0 | 0 | 0 | 0 | 0 | 2594.16 | 24.8 |
|  | 1.3. Settlement and collection of planulae | 595.56 | 24 X 2 people | 11.90/6.94* | 452.16 | 0 | 0 | 0 | 0 | 0 | 0 | 1047.72 | 10 |
| **2. Maintenance** |  | 257.54 | 3 X 1 person x 24 weeks | 6.94 | 499.68 | 0 | 0 | 0 | 0 | 0 | 0 | **757.22** | **7.2** |
| **3. Feeding regime** |  |  |  |  |  |  |  |  |  |  |  | **1000.9** | **9.6** |
|  | 3.1 Control treatment | 0 | - | - | 0 | 0 | 0 | 0 | 0 | 0 | 0 | 0 | 0 |
|  | 3.2 600 nauplii/L | 0.15 | 2 X 1 person x 24 weeks | 6.94 | 333.12 | 0 | 0 | 0 | 0 | 0 | 0 | 333.27 | 3.2 |
|  | 3.3 1800 nauplii/L | 0.46 | 2 X 1 person x 24 weeks | 6.94 | 333.12 | 0 | 0 | 0 | 0 | 0 | 0 | 333.58 | 3.2 |
|  | 3.4 3600 nauplii/L | 0.93 | 2 X 1 person x 24 weeks | 6.94 | 333.12 | 0 | 0 | 0 | 0 | 0 | 0 | 334.05 | 3.2 |
| **4. Transplantation** |  | 228.57 | 4 x 4 people | 11.90/6.94* | 150.72 | 4 | 111.11 | 14 | 133.33 | 1 | 436.51 | **860.24** | **8.2** |
| **5. *in situ* monitoring** |  | 3.17 | 1.5 X 2 people X 7 trips | 11.90/6.94* | 395.64 | 0 | 0 | 14 | 133.33 | 7 | 3055.56 | **3587.70** | **34.3** |
|  |  |  |  |  |  |  |  | Grand Total | | | | 10467.75 |  |
|  |  |  |  |  |  |  |  | *Ex situ* production cost for 288 coral plugs | | | | 6019.81 |  |
|  |  |  |  |  |  |  |  | *Ex situ* production cost/coral | | | | 20.90 |  |
|  |  |  |  |  |  |  |  | Cost/coral (51% survival) | | | | 40.98 |  |
|  |  |  |  |  |  |  |  | Cost/coral transplanted (128 coral plugs) | | | | 81.78 |  |
|  |  |  |  |  |  |  |  | Cost (54% survival) | | | | 151.44 |  |

Cost estimates of producing 288 plugs with live *Pocillopora damicornis* juveniles under four ex situ feeding regimes (0, 600, 1800, 3600 nauplii/L) for 24 weeks, followed by the transplantation of 128 coral plugs and subsequent monitoring for 24 weeks. Personnel involved in the study corresponded to skill levels 2 and 3 in Edwards et al. (2010), adopting local hiring rates in the National University of Singapore (NUS). The costs for SCUBA gear hire, air tank and boat were estimated based on local commercial rates. The monthly rental rate for aquaria facilities (18 m^2^) in the Tropical Marine Science Insitute, St Johns Island was US$22.35/m^2^. Mean survival rates across the treatments were used for the calculation of cost effectiveness at the end of each phase. All published rates are correct as of September 2013, and all costs were estimated in Singapore Dollars (S$) prior to conversion to US$ at the rate of S$ 1.26 = US$ 1.

* One skill level 2 and one level 3 personnel were involved in this activity.
